# A Macrocycle-Mediated Protein Cage

**DOI:** 10.1021/acsmacrolett.4c00656

**Published:** 2024-11-26

**Authors:** Ronan J. Flood, Aurélien Thureau, Peter B. Crowley

**Affiliations:** † SSPC, Science Foundation Ireland Research Centre for Pharmaceuticals, School of Biological and Chemical Sciences, University of Galway, University Road, Galway H91 TK33, Ireland; ‡ Synchrotron SOLEIL, L’Orme des Merisiers, Saint-Aubin BP 48, 91192 Cedex Gif-sur-Yvette, France

## Abstract

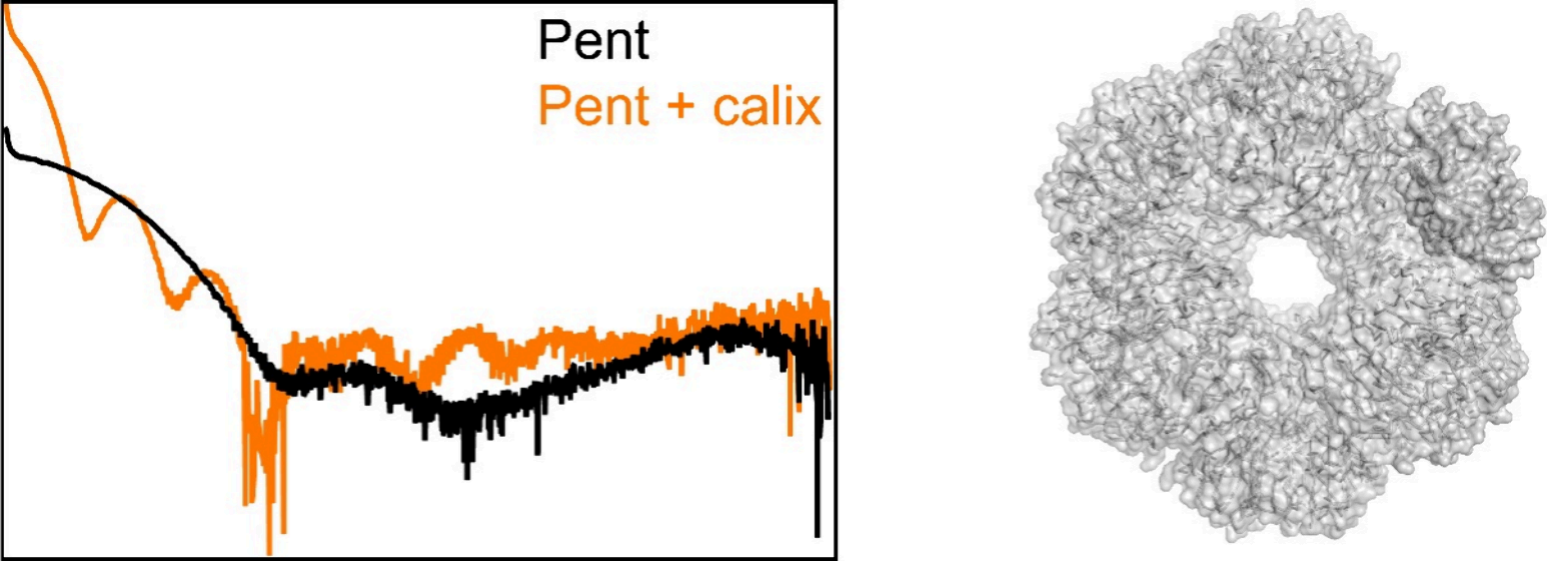

Engineered protein cages are of great interest considering their diverse applications in delivery and catalysis. Here, we describe macrocycle-triggered icosahedral cage assembly of a designed β-propeller. Cage assembly was evidenced by small-angle X-ray scattering and X-ray crystallography.

Protein cages are widespread in nature, serving as molecular containers and nanoreactors for nucleic acids (e.g., viral capsids), enzymes (e.g., bacterial microcompartments), metal ions (e.g., ferritin), and small molecules (e.g., lumazine synthase).^1,2^ There is great interest in engineering protein cages for diverse applications in delivery, imaging, catalysis, and reaction templating. Considering their appeal, numerous methods of controlling protein cage assembly^3−5^ as well as repurposing natural cages are in development.^6,7^

Pentagonal proteins, which cannot adopt planar tessellations, are good candidates for forming pseudospherical enclosed arrangements such as dodecahedra or icosahedra. Lumazine synthase is a widely studied example. The *Bacillus subtilis* protein forms pentamers that assemble into a dodecahedron of ∼15 nm diameter (e.g., PDB 1RVV).^8^ Engineered variants of lumazine synthase with enlarged icosahedral assemblies and repurposed cavities for accommodating non-natural guests are well established (e.g., PDB 7A4I).^9^ MhpD, an *Escherichia coli* hydratase, is another pentamer that naturally forms a dodecahedron with ∼20 nm diameter (PDB 2WQT).^10^ An engineered pentameric variant of PduA, a *Salmonella typhimurium* microcompartment shell protein, self-assembles into a 13 nm diameter dodecahedron (PDB 5HPN).^7^ The *Bacillus subtilis* stressosome (microcompartment) relies on pentameric protein RsbS that forms a ∼16 nm diameter icosahedron (PDB 6JHK).^11^ Recent advances in protein engineering are providing remarkable examples of *de novo* icosahedral assemblies. Two-component icosahedral complexes have been generated from engineered protein pairs that form, for example, 12 pentamers and 30 dimers (PDB 5IM4),^4^ while TIP60 is a fusion protein of a pentamer and a dimerizing coiled-coil (PDB 7EQ9).^12^ Ico8, is another engineered fusion protein, in this case, combining a trimeric protein with a pentamer forming coiled-coil.^13^

A simplified approach to cage assembly, independent of protein engineering, may further advance research in this field. Commercially available macrocycles, capable of binding protein surfaces and mediating assembly, are attractive candidates for cage formation. The ability to trigger^14^ cage assembly by adding a macrocycle may be advantageous with respect to labor-intensive protein engineering. Synthetic macrocycles can mediate porous protein assemblies in the solid state, including cage-like structures (e.g., space group *F*432).^14−17^ The anionic sulfonato-calix[n]arenes (**sclx**_**n**_) are particularly useful mediators. At least four crystal forms of the 6-bladed β-propeller *Ralstonia solanacearum* lectin (RSL) occur in the presence of the 1.5 kDa **sclx**_**8**_.^17,18^ These RSL–**sclx**_**8**_ polymorphs have solvent contents (porosities) ranging from 36 to 66% and the macrocycle acts as a molecular glue in varying degrees. Considering the packing limitations of a *C*_5_ object, we turned our attention to a pentameric protein. *Pent* is a cationic (p*I* ∼ 8), designed β-propeller pentamer that binds one N-acetyl-d-glucosamine (GlcNAc) per monomer (Figure 1). Pent does not appear to form icosahedral assemblies in the solid state (*c.f.* PDB entries 5C2N, 8R3D).^19,20^ Previously, we reported the 1:1 complex between Pent and **sclx**_**8**_.^20^ The cavity at the wide end of the Pent toroid accommodates a single **sclx**_**8**_ in a symmetry-mismatched multivalent complex. Now, we report the icosahedral assembly (a discrete cage) of Pent and **sclx**_**8**_ as characterized by SAXS and X-ray crystallography.

**Figure 1:**
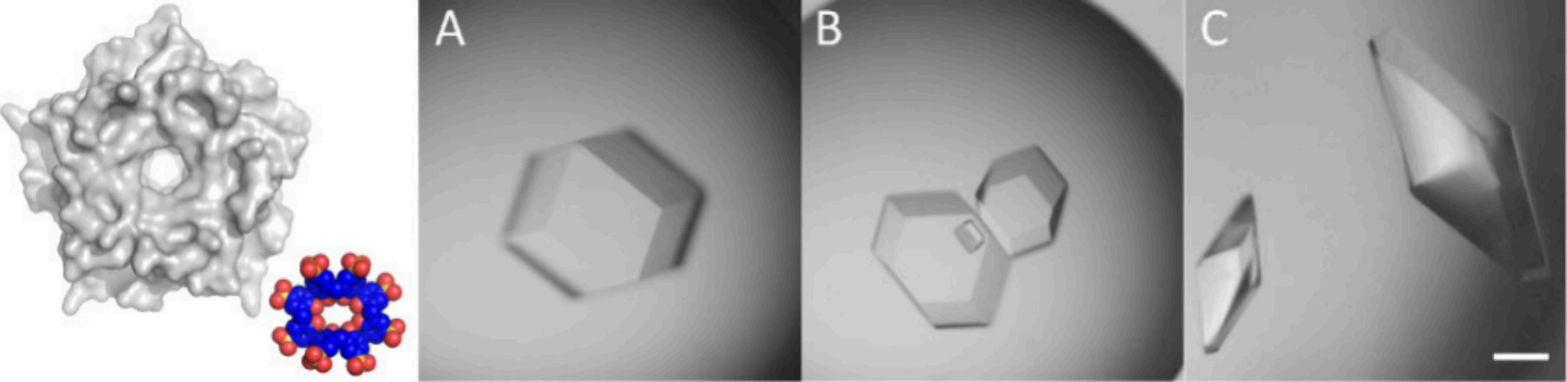
Surface and sphere representations of Pent and **sclx**_**8**_ (an extended conformation with carbon, sulfur, and oxygen in blue, yellow and red) drawn to scale and representative cocrystals of Pent with (A,B) **sclx**_**8**_ or (C) **sclx**_**6**_. The scale bar is 100 μm.

Previously, we obtained two cocrystal forms of the Pent–**sclx**_**8**_ complex in conditions comprising PEG precipitants with or without salt/buffer. In these structures (space groups *P*4_3_2_1_2 and *P*12_1_1) the macrocycle is positioned in the toroid cavity of Pent. Enclosed within this cavity the calixarene has minimal molecular glue activity and the protein assembly (crystal packing) is nonporous.^20^ Here, to obtain alternative crystal forms we further tested crystallization at high ionic strength (*I*) and calixarene concentrations ≥10 mM (Figure 1, Table S1). Crystals grew at *I* > 2, in the pH 4–7 range only in the presence of **sclx**_**8**_ (or **sclx**_**6**_).

Large rhombic crystals were obtained at 2 mM protein and 20 mM **sclx**_**8**_ in ∼1 M ammonium sulfate and 0.1 M buffer, either sodium acetate at pH 5.6 (Figure 1A) or sodium malonate at pH 5–7. Crystals also grew using a simple precipitant/buffer system such as 1.0–1.5 M sodium citrate pH 6 or sodium malonate pH 7 (Figure 1B). Irrespective of the crystallization condition, the diffraction data extended to 3.4 Å resolution only. A satisfactory solution was obtained in PHASER (see Methods) in space group *I*23 with an asymmetric unit comprising one pentamer (translation function Z score, TFZ = 13.0, log-likelihood gain LLG = 132.3). Integration in alternative cubic lattice systems was tested but structure solution was unsuccessful. The limited data resolution and the poor map quality prohibited detection of the macrocycle, but the data were sufficient to deduce the icosahedral assembly of Pent (Figure 2). The high porosity of the crystals with ∼70% solvent content (cavity diameter of ∼7 nm) may be a contributing factor to the poor diffraction. Lys33, highly exposed on the Pent vertices, is located close to the 2- and 3-fold symmetry axes of the icosahedral assembly suggesting that the anionic calixarene can glue these sites.

**Figure 2:**
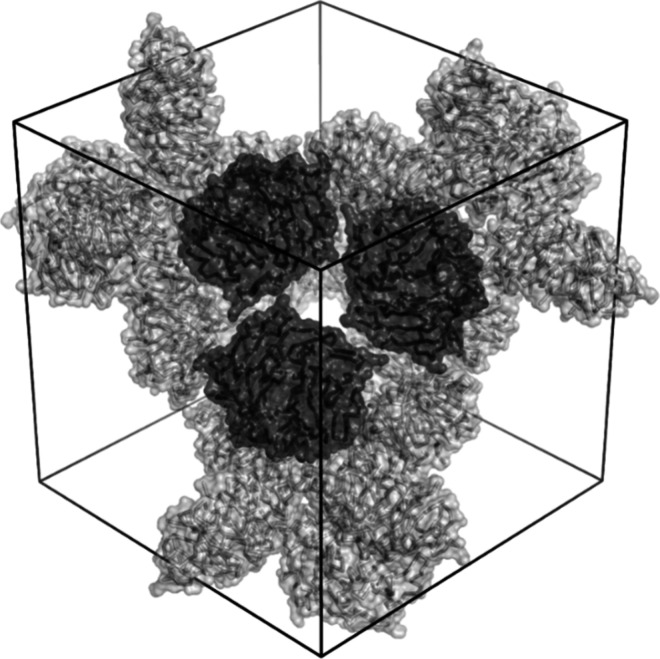
Pent–**sclx**_**8**_*I*23 unit cell, with proteins shown as gray surfaces. A 3-fold rotational axis is highlighted in dark gray. The unit cell contains two copies of the cage, with one complete cage in the center and four quarters of the cage arranged on the corners. The unit cell length is 135 Å.

A crystal structure of Pent and the *C*_6_-symmetric **sclx**_**6**_ provides supporting evidence for this inference. Like the Pent–**sclx**_**8**_ cocrystals, Pent–**sclx**_**6**_ was cocrystallized in 1.2 M ammonium sulfate and 0.1 M sodium malonate pH 7 (Figure 1C). These crystals diffracted to 1.7 Å resolution, and the structure was solved in space group *C*222_1_ with an asymmetric unit comprising one Pent molecule. Four **sclx**_**6**_ ligands are evident in the unbiased electron density map (Figure S1). Adopting the “double partial cone” conformation, **sclx**_**6**_ binds the solvent exposed loop residues 30–34 at three of the Pent subunits. Consistent with our hypothesis, multiple noncovalent bonds are formed between the sulfonato-calixarene and up to 5 residues including Lys33 (and/or Lys22) from two protein subunits of two Pent molecules (Figure 3). The macrocycle position at the pentamer vertex suggests that **sclx**_**8**_ might form similar interactions in assembling the icosahedron (Figure 2). Importantly, crystals of Pent in the absence of **sclx**_**n**_ did not yield any cage-like assemblies. Pure Pent crystals grow in PEG-containing conditions (PDB 8R3D), similar to the parent protein (PDB 5C2N).^19,20^ In this work, a new crystal of pure Pent (obtained in Jena JCSG++ HTS condition A12, 20% PEG 3350, 0.2 M potassium nitrate) diffracted to 1.7 Å resolution and the structure was solved in space group *P*12_1_1 with an asymmetric unit comprising two Pent molecules (Figure S2).

**Figure 3:**
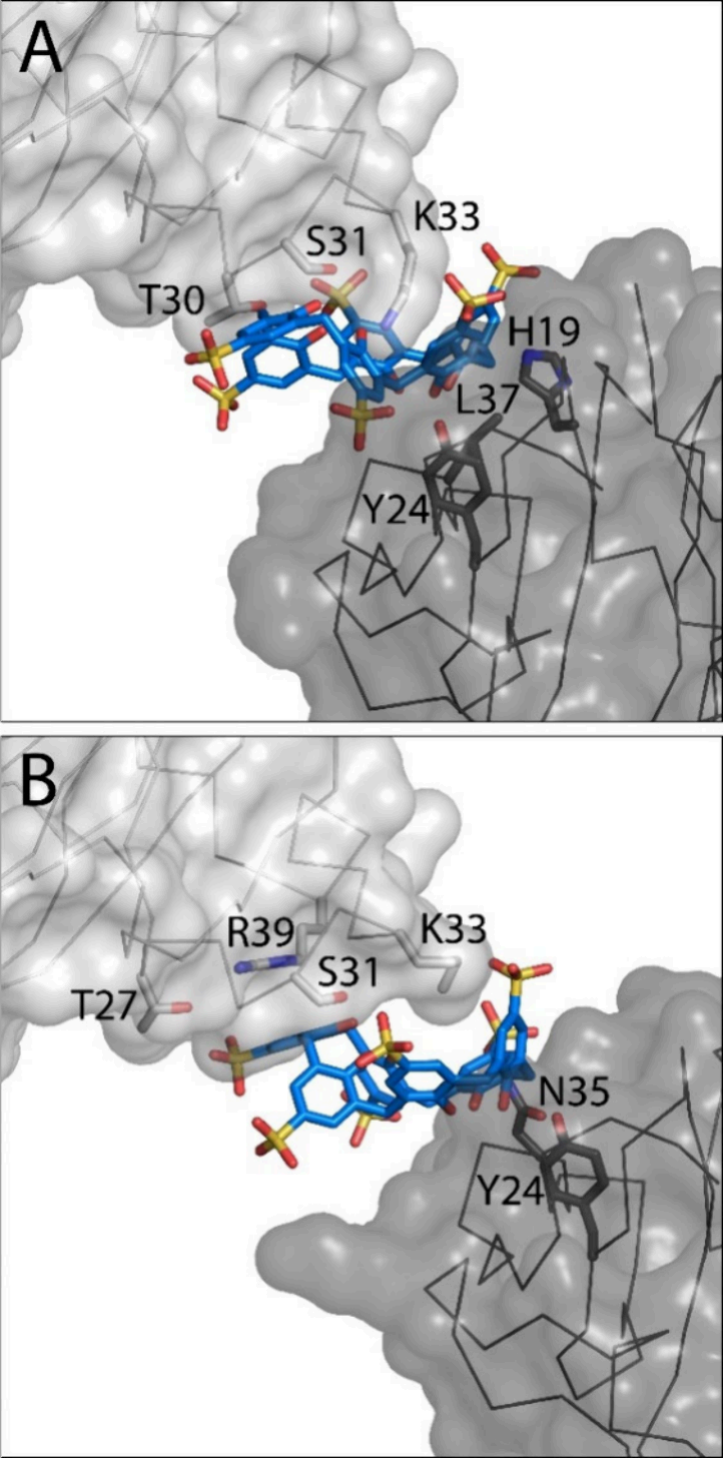
Details of two Pent–**sclx**_**6**_–Pent crystallographic interfaces, highlighting the calixarene glue activity at Lys33. Proteins shown as C^α^ trace and transparent surfaces with interfacing side chains as sticks. Symmetry mates indicated in dark gray.

To gain more insight into the assembly possibilities, we characterized pure Pent and Pent–**sclx**_**8**_ mixtures by SAXS (Figures 4, S3, and S4). A high salt buffer was used, thereby emulating the crystallization conditions. Table 1 lists the radius of gyration (*R*_g_), maximum particle size (*D*_max_), Porod volume (*V*) and the molecular mass of each sample. The SAXS data for pure Pent in 0.8 M sodium citrate pH 6, confirmed a monodisperse sample with negligible assembly in the 0.5–4 mM protein concentration range (Figures 4A and S3). Data analysis in PRIMUS (see Methods) revealed a *R*_g_ of ∼19 Å and a calculated molecular mass of ∼23 kDa, in agreement with the computed scattering of the ∼26 kDa Pent. The scattering curve was altered significantly by the addition of 2 mM **sclx**_**8**_ (Figure 4B). The ∼2-fold larger *R*_g_ (37 Å) is consistent with **sclx**_**8**_-mediated dimerization of Pent, as observed previously (PDB 8R3C).^20^ However, the scattering data did not fit the computed scattering of any crystallographic dimer (Figure S4). Attempts to interpret the data with computed scattering from mixtures of Pent and oligomers such as tetramers and hexamers, yielded poor fits. These data, suggesting sample heterogeneity (due to insufficient calixarene for complete assembly) are nevertheless consistent with higher order assembly. In contrast, the SAXS data for 2 mM Pent and 20 mM **sclx**_**8**_ were striking (Figure 4C) and corresponded to an icosahedral assembly with *R*_g_ ∼ 60 ± 15 Å, consistent with the crystallographic dimension (Figure 2). Data analysis in PRIMUS revealed a large confidence interval (264–412 kDa) for the molecular mass, hindering detailed analysis. Assuming 0, 1, or 2 **sclx**_**8**_ per Pent yields icosahedral assembly masses of 312, 330, or 348 kDa, such values are within the observed range (Table 1). The agreement between the experimental SAXS scattering curves and the computed scattering of the crystallographic assembly suggests that the discrete icosahedral cage can occur both in solution and in the solid state. However, we cannot exclude that the icosahedral assembly represents a fraction of the sample. Indeed, size exclusion chromatography at both low and high (Figure S5) ionic strength yielded Pent only and not the assembly. These data suggest that the icosahedral assembly is mediated by weak Pent–**sclx**_**8**_ interactions (dissociation and separation in the gel preventing assembly).

**Figure 4:**
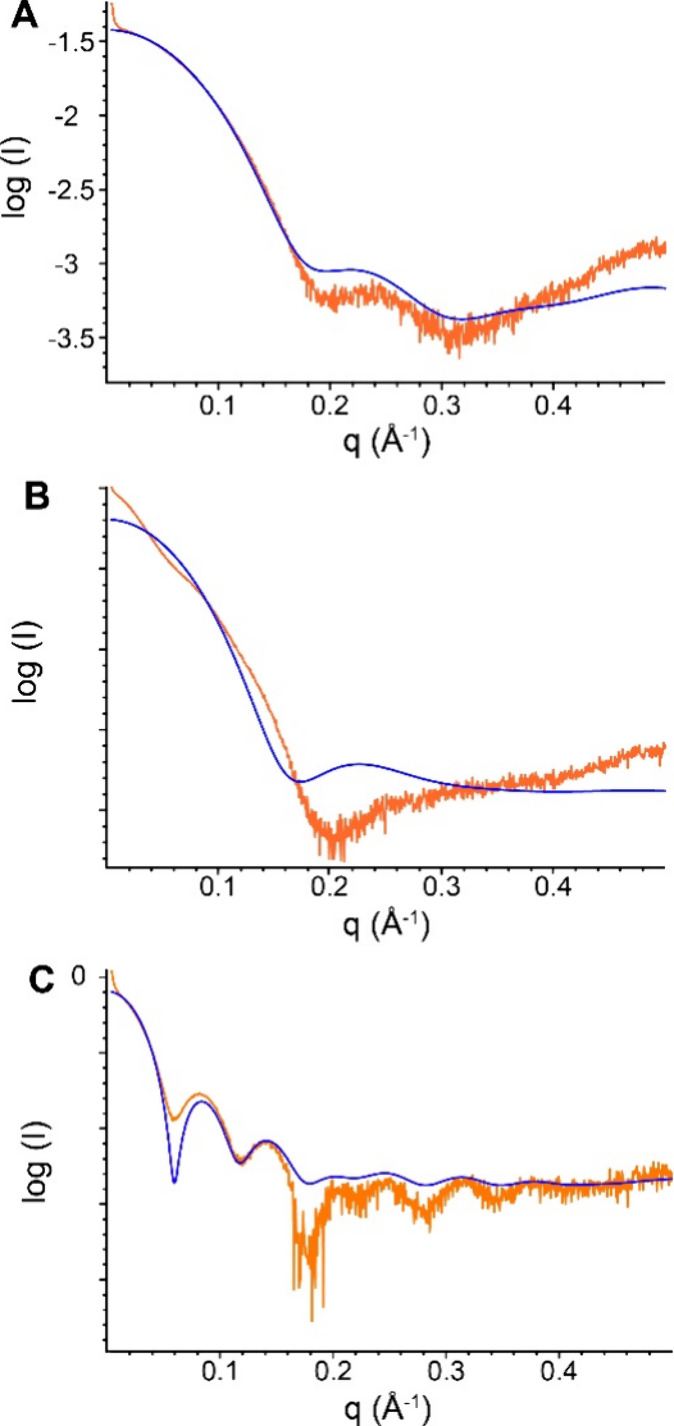
Logarithmic SAXS profiles (orange) of 2 mM Pent with (A) 0, (B) 2, or (C) 20 mM **sclx**_**8**_. Blue curves are the CRYSOL-computed scattering of (A) Pent, (B) a Pent dimer (PDB 8R3C), and (C) the icosahedral assembly (Figure 2).

**Table 1:** SAXS-Derived Parameters for Pent and Pent–**sclx**_**8**_ Mixtures

Sample	Pent	Pent + 2 mM **sclx_8_**	Pent + 20 mM **sclx**_**8**_
*R*_g_ (Å)	19.4 ± 1.4	37.0 ± 0.7	60.5 ± 14.9
*P*(*r*) analysis			
*D*_max_ (Å)	62.8	143.8	235.2
*R*_g_ (Å)	19.2	38.7	60.5
Porod Vol. (Å^3^)	35,700	56,100	707,000
Mol. mass (kDa)			
Bayesian Inf	23	-	318
Bayesian Inf confidence interval	21–24	-	264–412
Theoretical mass	26	52	312

As a “molecular glue” **sclx**_**8**_ can mediate different types of solid-state porous protein assemblies.^14,17^ Here, **sclx**_**8**_ directed the assembly of a pentameric β-propeller into a discrete icosahedral cage, evidenced in solution (i.e., a closed oligomer as opposed to an extended or infinite assembly occurring in the solid state). Potential calixarene binding sites were revealed in the Pent–**sclx**_**6**_ crystal structure, obtained under similar conditions. Formation of the icosahedral assemblies was contingent on two factors; high ionic strength and high concentrations (20 mM) of **sclx**_**8**_. The dependence on high ligand concentrations could be exploited to yield stepwise triggering of protein assembly,^14^ where cage formation is delayed until the protein/ligand mixture reaches the required level. Such a stepwise process may prove useful for biomedical applications. Future work will extend the possibilities of macrocycle-mediated cage assembly.

## Experimental Methods

### Materials

GlcNAc (A0092), **sclx**_**6**_ (S0470), and **sclx**_**8**_ (S0471) were obtained from Tokyo Chemical Industry. Calixarenes were prepared as ∼100 mM stock solutions at pH ∼ 7 in water. Pent was produced as described.^20^ All experiments were performed with GlcNAc-bound protein.

### Crystallization Trials

Hanging drop vapor diffusion experiments were performed in 24 well Greiner plates at 20 °C with mixtures of 2 mM Pent, 10 mM GlcNAc, and 0–20 mM **sclx**_**8**_ or 0–50 mM **sclx**_**6**_. The conditions comprised 0.5–2.5 M ammonium sulfate and 0.1 M buffer, either sodium citrate pH 4.0, sodium acetate pH 4.6–5.6, sodium malonate pH 4.0–7.0, or Tris-HCl pH 7.0. Pent–**sclx**_**8**_ trials were also performed with 0.5–1.5 M sodium malonate pH 7.0 or 0.5–1.5 M sodium citrate pH 6.0.^21^

### X-ray Data Collection and Structure Determination

Crystals were transferred to reservoir solution supplemented with 25–30% glycerol and cryo-cooled in liquid nitrogen. Diffraction data were collected at 100 K on beamline PROXIMA-2A, SOLEIL synchrotron (France) with an Eiger X 9 M detector (Table S2). Data processing relied on the autoPROC pipeline,^22^ with integration in XDS,^23^ scaling in AIMLESS^24^ and merging in POINTLESS.^25^ Crystal pathologies were assessed in phenix.Xtriage.^26^ Structures were solved by molecular replacement in PHASER^27^ using PDB 8R3D as the search model. The coordinates for **sclx**_**6**_ (ligand id FWQ) and GlcNAc (ligand id NDG) were added in Coot.^28^ Iterative model building and refinement were performed in Coot and phenix.refine.^26^ The Pent–**sclx**_**6**_ structure and structure factor amplitudes were deposited in the Protein Data Bank (id 9FRO) after validation in MolProbity.^29^ The statistics are reported in Table S2.

### Size Exclusion Chromatography

SEC experiments were performed at room temperature on an Äkta Purifier with a Superdex 75, GL 10/300 column (GE Healthcare).^14^ Filtered buffers (0.8 M sodium citrate, 5 mM GlcNAc pH 6.0 or 20 mM potassium phosphate, 100 mM sodium chloride, 2 mM GlcNAc pH 6.0) were pumped at a constant flow rate of 0.5 mL/min. Samples (300 μL) containing 0.2–0.5 mM protein plus 0–20 equiv **sclx**_**8**_ were centrifuged at 14,680 rpm for 10 min immediately prior to injection. Elution was monitored at 280 nm.

### SAXS Data Collection and Analysis

Pent was exchanged into 0.8 M sodium citrate, 5 mM GlcNAc, pH 6.0 via size exclusion chromatography. SAXS data were collected at the SWING beamline (SOLEIL synchrotron) using the direct injection mode and the automated sample changer.^30^ To characterize the protein only, samples of 0.5, 1, 2, and 4 mM Pent were measured (Table 1, Figure S3). To characterize the complex, samples of 2 mM Pent and 0, 2, or 20 mM **sclx**_**8**_ were measured. The scattering images were processed with masking, azimuthal averaging, curve selection and buffer signal subtraction in FOXTROT (SOLEIL Synchrotron). SAXS data plots were generated in PRIMUS.^31^ The forward scattering and radius of gyration (*R*_g_) were calculated from the Guinier approximation.^32^ The maximum particle size (*D*_max_) was determined from the pair distribution function computed by GNOM^33^ in PRIMUS. The molecular weight was estimated by Bayesian inference with four methods MoW,^34^ Size&Shape,^35^ Vc^36^ and MMQp,^37^ as implemented in Atsas.^38^ CRYSOL^39^ was used to calculate the scattering of Pent and different oligomers. OLIGOMER was used to determine the volume fraction of the species in the samples.

## Supporting Information Available

Additional details, including Figures S1–S5 and Tables S1 and S2 (PDF)
