# Supplementary material for: A Macrocycle-Mediated Protein Cage
Source: ACS Macro Lett. 2024 Nov 26;13(12):1686–90. doi: 10.1021/acsmacrolett.4c00656 (PMC11656731; doi:10.1021/acsmacrolett.4c00656)
Supplement: Supplementary file 1 — mz4c00656_si_001.pdf [file mz4c00656_si_001.pdf]

Supporting Information

A Macrocycle-mediated Protein Cage

Ronan J. Flood,[a] Aurélien Thureau,[b] Peter B. Crowley*,[a]

[a]SSPC, Science Foundation Ireland Research Centre for Pharmaceuticals, School of Biological and Chemical Sciences, University of Galway, University Road, Galway H91 TK33, Ireland.

[b]Synchrotron SOLEIL, L'Orme des Merisiers, Saint-Aubin BP 48, 91192 Gif-sur-Yvette Cedex, France.

*Correspondence to: peter.crowley@universityofgalway.ie +353 91 49 24 80

Keywords: β-propeller; calixarene; crystal; icosahedral assembly, SAXS

Contents

Table S1	Crystallization conditions and structure properties.	S3
Table S2	X-ray data collection, processing, and refinement statistics	S4
Figure S1	Electron density map showing **$sclx_6$** complexing Pent	S5
Figure S2	Pent only crystal dimers	S6
Figure S3	SAXS data for Pent only	S7
Figure S4	SAXS data for Pent plus 2 eq **$sclx_8$** and oligomer modelling	S8
Figure S5	SEC of Pent in the presence or absence of **$sclx_8$**	S9

Table S1. Crystallization conditions and structure properties.

Structure	Precipitant	Buffer	pH	Space Group	*a* × *b* × *c* (Å)	Res. (Å)	Pore Ø (nm)[a]	PDB id
Pent – $\mathbf{sclx_8}$	0.9-1.3 M $(NH_4)_2SO_4$	acetate	4.6-5.6	*I*23	135^3	3.4	6.9	-
		malonate	4.0-7.0					
	1.5-1.8 M $(NH_4)_2SO_4$	Tris-HCl	7.0					
	1.0-1.5 M Na citrate	-	5.0					
	1.0-1.5 M Na malonate	-	7.0					
Pent – $\mathbf{sclx_6}$	1.2 M $(NH_4)_2SO_4$	malonate	7.0	$C222_1$	56 x 93 x 112	1.7	1.8	9FRO

[a]Diameter of widest pore, calculated in MAP_CHANNELS.

Table S2. Crystallization conditions and X-ray data collection, processing, and refinement statistics for Pent – **sclx**$_n$ crystals.

Structure	Pent – **sclx**$_6$	Pent – **sclx**$_8$
	Crystallization conditions	
**Protein (mM)**	2	
**sclx$_n$ (mM)**	10	20
**Precipitant**	1.2 M $(NH_4)_2SO_4$	
**Buffer (0.1 M)**	sodium malonate	sodium acetate
**pH**	7.0	5.6
	Data collection[a]	
**Light source**	SOLEIL, PROXIMA-2A	
**Wavelength (Å)**	0.98011	
**Space group**	*C*222$_1$	*I*23
**a, b, c (Å)**	55.570<br>92.689<br>112.376	134.528<br>134.528<br>134.528
**Resolution (Å)**	56.19-1.71 (1.74-1.71)	67.26-3.35 (3.40-3.35)
**# reflections**	387365 (18457)	227701 (12919)
**# unique reflections**	31640 (1593)	6003 (306)
**Multiplicity**	12.2 (11.6)	37.9 (42.2)
**I/σI**	12.4 (2.1)	17.2 (2.8)
**Completeness (%)**	100.0 (100.0)	100.0 (100.0)
**R_{meas}[b] (%)**	12.9 (150.5)	15.2 (178.4)
**R_{pim}[c] (%)**	3.7 (43.9)	2.5 (27.4)
**$CC_{1/2}$**	99.8 (77.9)	100.0 (84.5)
**Solvent content (%)**	55	70
	Refinement	
**R_{work}**	19.0	-
**R_{free}**	21.6	-
**rmsd bonds (Å)**	0.005	-
**rmsd angles (°)**	0.775	-
	**# molecules in asymmetric unit**	
**Pent**	1	1
**sclx$_n$**	4	-
**GlcNAc**	4	-
**Water**	135	-
**Avg. B-factor (Å^2)**	27.8	-
**Clashscore**	2.0	-
	**Ramachandran analysis,[d] % residues in**	
**favoured regions**	98.23	-
**allowed regions**	1.77	-
**PDB code**	9FRO	-

[a]Values in parentheses correspond to the highest resolution shell [b]Rmeas = ∑hkl √(n/n-1)∑I |Ii(hkl)-⟨I(hkl)⟩ |/∑hkl ∑i Ii(hkl); [c]Rpim = ∑hkl √(1/n-1)∑ n i=1 |Ii(hkl)- |/∑hkl ∑i Ii(hkl); [d] ⟨I(hkl)⟩ Calculated in MolProbity.

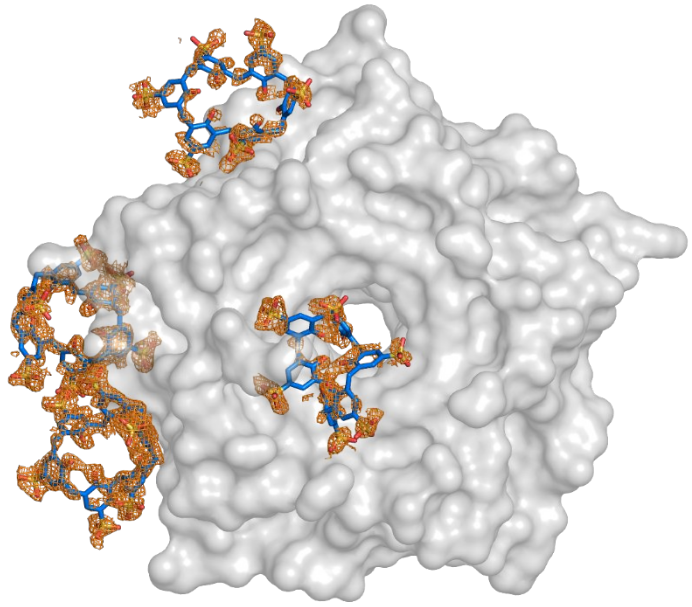

Figure S1. The unbiased 2Fo - Fc electron density maps, contoured at 1.0 σ (orange mesh), reveal the locations of **sclx$_6$** on Pent.

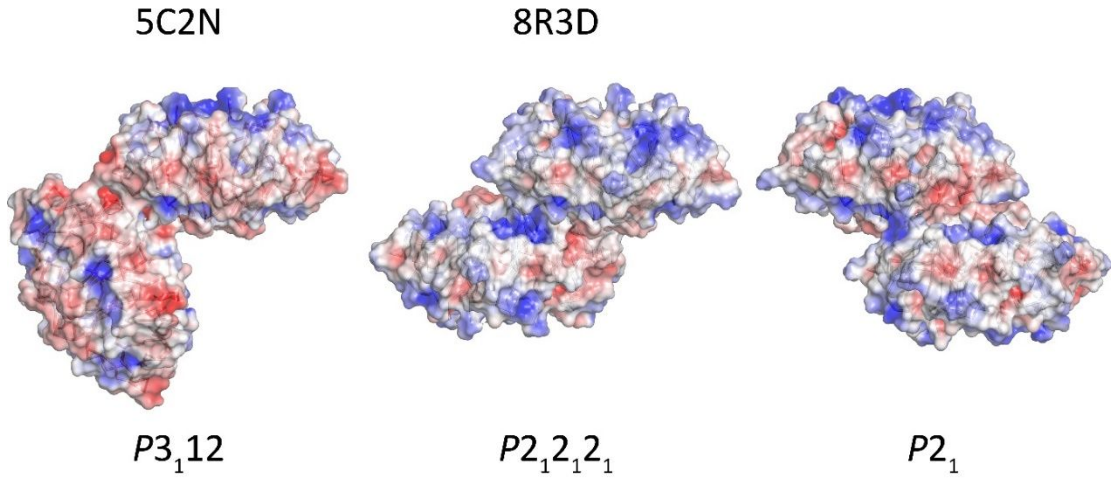

Figure S2. Dimers of Pent in protein only crystal structures include published examples PDB 5C2N and 8R3D.

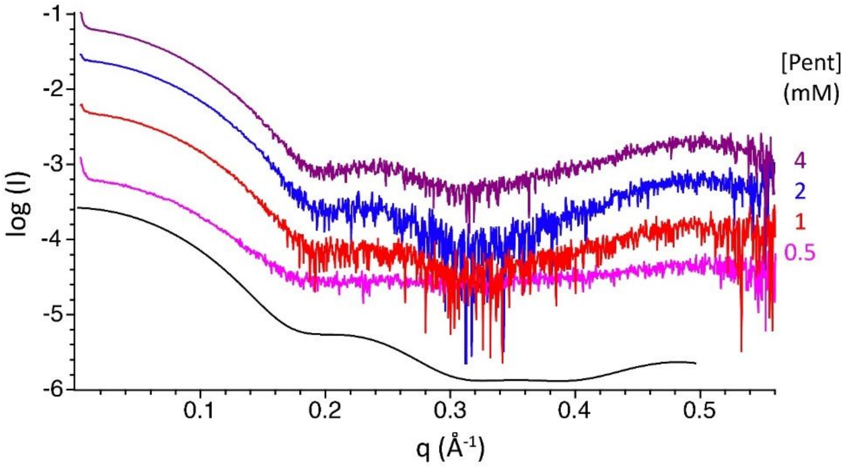

Figure S3. SAXS analysis of pure Pent in 0.8 M sodium citrate, 5 mM GlcNAc, pH 6.0 reveals a monodisperse species, in good agreement with the Pent crystal structure (PDB 8R3D, computed scattering in black).

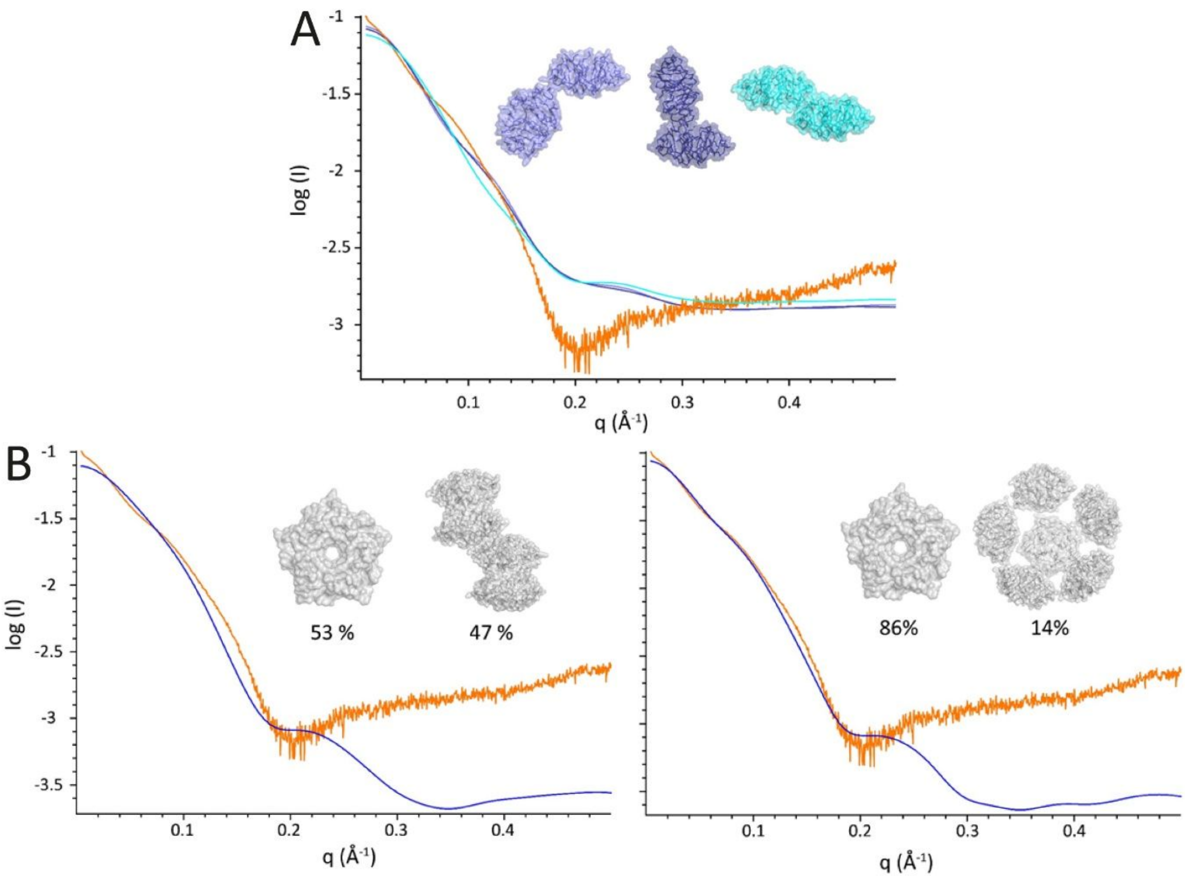

Figure S4. SAXS profile of 2 mM Pent + 2 mM **sclx$_8$** (orange) and CRYSOL computed scattering from **(A)** different crystallographic dimer models or **(B)** mixtures of Pent and tetramer (left) or hexamer (right) assemblies, calculated in OLIGOMER.

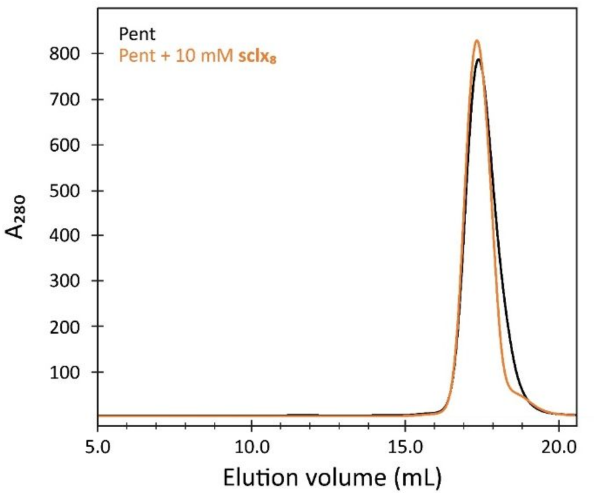

Figure S5. Size exclusion chromatograms of Pent in the presence (orange) or absence (black) of **sclx$_8$**. Elution buffer 0.8 M sodium citrate, 5 mM GlcNAc, pH 6.0.
